# Radiotherapy-based multimodal treatment in advanced NSCLC with spinal infiltration: a case series of seven unfavorable patients

**DOI:** 10.1007/s12672-026-05094-x

**Published:** 2026-04-29

**Authors:** Linda Agolli, Bastian Eul, Diethard Prüfer, Sebastian Harth, Stefan Gattenlöhner, Daniel Habermehl

**Affiliations:** 1 Department of Radiation Oncology, Giessen University Hospital, Justus-Liebig-University Giessen, Giessen, Germany; 2 Department of Internal Medicine, Justus-Liebig-University Giessen, Giessen University Hospital, Giessen, Germany; 3 Department of Thoracic Surgery, Kerckhoff Heart and Thorax Center, Bad Nauheim, Germany; 4 Department of Diagnostic and Interventional Radiology, Justus-Liebig-University, Giessen University Hospital, Giessen, Giessen, Germany; 5 Institute of Pathology, Justus-Liebig-University Giessen, Giessen University Hospital, Giessen, Germany

**Keywords:** NSCLC, Spine invasion, Radiotherapy, Surgery, Prognosis

## Abstract

**Background:**

Locally advanced non-small cell lung cancer (LA-NSCLC) with spinal invasion presents a rare but challenging clinical scenario. Radical surgical approaches, though potentially curative, are often not feasible due to comorbidities, advanced age, poor performance status or technical inoperability. In this setting, the optimal multimodal treatment strategy including radiotherapy (RT) and systemic treatment remains to be fully defined.

**Methods:**

We retrospectively analyzed seven patients with stage III/IV NSCLC and radiologically confirmed spinal invasion treated between 2023 and 2025. Treatment modalities included radiotherapy, systemic therapy, and surgical procedures. Patients were retrospectively stratified using the prognostic scoring system proposed by Lei et al. to assess clinical outcomes by risk group.

**Results:**

Four patients were classified as low-risk (score 4–5), and three as intermediate-risk (score 6–7); no patients fell into the high-risk category. Two low-risk patients showed favorable outcomes, including long-term survival and disease stability, while two succumbed to local progression. All intermediate-risk patients died within 5–13 months due to disease progression. Radical surgery was not feasible in any case. Timely treatment initiation and multidisciplinary care were associated with improved outcomes in selected patients.

**Conclusion:**

RT-based multimodal treatment appears feasible in selected unfavorable patients with advanced NSCLC and spinal infiltration who are not candidates for radical surgery. The prognostic score can help in risk stratification, though timely therapeutic decision-making is equally critical. However, due to the small sample size, the impact on survival remains uncertain and requires validation in larger studies.

## Introduction

Locally advanced non-small cell lung cancer (LA-NSCLC) is a significant clinical challenge, characterized by an often-dismal prognosis. Spinal infiltration in this setting is a serious complication that can cause severe pain and neurological sequelae, significantly impacting patient quality of life and survival [1]. Traditionally considered unresectable and with a poor prognosis, the treatment of LA-NSCLC with spinal invasion has undergone a remarkable evolution over the years. Initially, the surgical approach was limited, but with the refinement of surgical techniques and the integration of multimodal therapies, including induction chemoradiation (CRT) therapy followed by radical surgical resection en bloc, significant improvements in local disease control and long-term survival have been observed in selected patients [2].

However, the management of these patients remains complex, with significant postoperative morbidity associated with extensive surgery. Moreover, many patients are inoperable due to comorbidity and advanced age. The advent of immunotherapies, such as immune checkpoint inhibitors, has further expanded the therapeutic options for locally advanced and metastatic NSCLC and has shown promise even in the presence of metastases to sensitive sites such as the central nervous system [3].

Radiation therapy (RT) in combination with chemotherapy is the preferred treatment for inoperable LA-NSCLC. Nevertheless, dose prescription is restricted in the intraspinal tumor region due to spinal cord and nerve constraints [4–7]. The existing literature is scarce, primarily consisting of small retrospective surgical series, with only occasional reports on radiotherapy [8–12]. In fact, there is a lack of data regarding optimal RT dosing strategies for these complex cases.

The presentation of a clinical case series of locally advanced NSCLC with spinal infiltration may help to better delineate optimal treatment strategies with focus to RT and achievable outcomes in the era of combination therapies.

## Methods

We retrospectively reviewed the medical records of adult patients diagnosed with LA-NSCLC and intraspinal invasion from 2023 to 2025. All patients had a histologically confirmed diagnosis and were staged with computed tomography (CT) of the thorax, positron emission tomography (PET)-CT and brain magnetic resonance imaging (MRI). Vertebral invasion was assessed by MRI to evaluate the extent of bone infiltration and the extent of foraminal and epidural involvement. Decision to treatment modality was validated in a multidisciplinary tumor board.

The aim of this study was to describe clinical outcomes and the feasibility of radiotherapy-based multimodal treatment in unfavorable patients with advanced NSCLC and spinal infiltration who were not candidates for radical surgical resection.

A score system was developed by Lei et al. to evaluate prognostic factors in patients with metastatic spinal invasion from NSCLC in order to support decision-making for surgical intervention in this high-risk population [13]. The scoring system ranged from 4 to 10 points and incorporated independent variables such as preoperative neurological status, presence of visceral metastases, and Eastern Cooperative Oncology Group (ECOG)-performance status (PS) to stratify patients into groups with significantly different survival expectations. According to the final score, 3 prognostic groups were defined: 4–5 points (most favorable survival), 6–7 points (intermediate survival rates), and 8–10 points (worse prognosis). We scored our patients retrospectively to evaluate prognosis (See Table 2).

**Table 1:** Prognostic score (according to Lei et al. [13]) and outcome

ECOG performance status							
Risk factors	Case 1	Case 2	Case 3	Case 4	Case 5	Case 6	Case 7
1–2	1	1	1	1	1	1	1
3–4							
Number of involved vertebrae							
1–2	1	1	2	1	2	2	2
≥3							
Visceral metastases							
No	1	2	1	2	2	1	1
Yes							
Time developing motor deficits							
< 14 days	1	3	1	3	1	1	1
≥ 14 days							
Score [13]	4 points	7 points	5 points	7 points	6 points	5 points	5 points
Outcome	Alive 2 years after RT	Local and distant PD during RT, emergency OP, death immediately after OP due to PD	Death during RT due to local PD	Death immediately after OP due to local and distant PD	Death during RT due to local progression	Death after local PD after CT and RT (no response)	Alive with SD
Survival from diagnosis	26 months	13 months	2 months	7 months	5 months	7 months	2 months

ECOG: Eastern Cooperative Oncology Group, OP: surgery, RT: radiotherapy, PD: progressive disease, CT: chemotherapy

### Patients and treatment characteristics

In the current case series, a total of seven patients with LA-NSCLC and spinal invasion were included in this retrospective case series. The median age was 64 years (range 56–75), with five males and two females. Most patients (6/7) had a smoking history and comorbidities. ECOG performance status ranged from 1 to 2.

Histologically, six patients had squamous cell carcinoma and one had adenocarcinoma. PD-L1 expression was positive in five cases (1–5%), negative in one, and unknown in one. A BRAF mutation was detected in one patient. Three patients presented with local recurrence after previous surgery, while four had newly diagnosed advanced NSCLC. Tumor stage ranged from cT1cN0M1a to T4N2M0, with frequent involvement of upper or lower lobes and direct spinal infiltration (including Pancoast tumor in one case). In our cohort, two patients had stage cM1a oligometastatic disease. However, both were selected for definitive thoracic radiotherapy with curative intent. In one case, the patient presented with pleural carcinomatosis but achieved a complete response during systemic therapy and was subsequently considered for curative radiotherapy. In the second case, a single subpleural metastasis was included within the radiation field, allowing delivery of a curative radiation dose. Patient and tumor characteristics are summarized in Table 1.

**Table 2:** Patients, tumor and treatment characteristics

Patients‘characteristics	Case 1	Case 2	Case 3	Case 4	Case 5	Case 6	Case 7
Age (years)	64	56	75	60	69	68	62
Gender	Male	Male	Male	Male	Male	Female	Female
ECOG-PS	1	1	2	1	2	2	2
Smoking history	Yes	No	Yes	Yes	Yes	Yes	Yes
Comorbidity	Yes	No	Yes	Yes	Yes	Yes	Yes
Tumor characteristics							
Histology	Squamous	Adenocarcinoma	Squamous	Squamous	Squamous	Squamous	Squamous
PDL1-status	Pos, 1%	Neg	Neg	Pos, 2%	Pos, 5%	Pos, 5%	Pos, ??
Driver mutation	No	BRAF-mutation	No	No	No	No	No
Postoperative local relapse	No	No	Yes	Yes	No	No	No
Primary diagnosed NSCLC	Yes	Progression after initial CR after 1 year	Postoperative local relapse 7 years after surgery	Postoperative local relapse 4 months after surgery	Yes	Yes	Yes
TNM Stage	T4N2M0	cT1cN0cM1a	rcT4rcN0cM0	rcT4rN2cM0	cT3cN2cM1a	cT3cN0cM0 (Pancoast)	cT4cN0cM0
Tumor location	LL left	LL right	LL left	LL right	UL right	UL right	UL right
Metastases at diagnosis	No	Pleural carcinomatosis (CR before RT)	No	No	Singular subpleural metastasis	No	No
Treatment characteristics							
Primary therapy	CT	Target therapy	Surgery	CT/IT	IT	CT	RT
Surgery	No	Yes (decompression because of PD during RT)	Yes (laminectomy before RT)	Yes (laminectomy because of PD after RT)	No	No	No
Systemic therapy	Carboplatin+ paclitaxel, Durvalumab - maintenance	Trametinib/ Dabrafenib	No	Carboplatin+ nab-Paclitaxel+ Nivolumab	Pembrolizumab	Cisplatin+ vinorelbine	No
Radiotherapy	Yes	Yes	Yes	Yes	Yes	Yes	Yes
Radiation volume	- Primary tumor+ involved nodes + Rib - SIP spinal canal	- Primary tumor+ involved nodes rib - involved vertebra (one) -SIP spinal canal	- Primary tumor+ involved nodes - involved vertebra (three)+ intraspinal tumor	- Primary tumor with soft tissue components - involved vertebra (one) + rib	- Primary tumor+ pleural metastasis - intraspinal soft issue tumor - involved vertebra (three)	- Primary tumor + involved nodes - involved vertebra (three)	- Primary tumor+ involved nodes - involved vertebra (three)
Radiation prescribed dose	− 66 Gy/33 fr − 59.4 Gy/33 fr - spinal cord < 47 Gy	-60 Gy/30 fr − 53,4 Gy/30 fr - spinal cord < 47 Gy	-56,25 Gy/25 fr (EQD2 59,8 Gy) − 45 Gy/25 fr - spinal cord ≤ 45 Gy	-55 Gy/20 fr (EQD2 59,8 Gy) − 40 Gy/20 fr - spinal cord < 45 Gy	-60 Gy/30 fr − 50 Gy/20 fr − 40 Gy/20 fr - spinal cord ≤ 45 Gy	-66 Gy/33 fr − 40 Gy/20 fr - spinal cord < 47 Gy	-60 Gy/30 fr − 48 Gy/30 fr - spinal cord < 45 Gy
Radiation technique	VMAT	VMAT	VMAT	VMAT	VMAT	VMAT	VMAT
Outpatient/inpatient	Inpatient	Inpatient	Inpatient	Inpatient	Inpatient	Inpatient	Inpatient
Toxicity CTC AE V5.0	Esophagitis G2 Pneumonitis G2	No tox	Dyspnea G2	Fatigue G1 Cough G1	Fatigue G2	No tox	No tox

NSCLC: non-small-cell lung cancer, RT: radiotherapy, OP: surgery, CT: chemotherapy, IT: immunotherapy, LL: lower lobe, UL: upper lobe, PD: progressive disease, CR, complete response, PDL1: programmed death-ligand 1, VMAT: volumetric modulated arch therapy, SIP: simultaneous integrated protection, EQD2: equivalent dose 2 Gy, CTC AE V5.0: Common Terminology Criteria for Adverse Events OV5.0

All seven patients received RT as part of a multimodal treatment approach with curative intent. RT was delivered using volumetric modulated arc therapy (VMAT) and administered in an inpatient setting due to the complexity of the disease and the need for close clinical monitoring. Target volumes included the primary lung tumor, involved lymph nodes, and areas of vertebral and/or intraspinal tumor extension, with adjacent structures such as ribs, pleura, or soft tissue components included when tumor infiltration was present. Prescribed radiation doses ranged from 40 to 66 Gy using different fractionation schedules (1.6–2.75 Gy per fraction), with dose selection individualized according to tumor extent and proximity to organs at risk, particularly the spinal cord. Spinal cord dose constraints were applied in all treatment plans, with maximum doses limited to ≤ 45–47 Gy depending on fractionation. Systemic therapy varied across patients and included platinum-based chemotherapy combined with immune checkpoint inhibitors (e.g., durvalumab, nivolumab), immunotherapy alone (pembrolizumab), or targeted therapy with dabrafenib and trametinib in one patient harboring a BRAF mutation. Three patients underwent spinal surgical procedures (laminectomy or decompressive surgery), performed either before radiotherapy or due to disease progression or neurological symptoms during or after RT. Radical surgical approaches such as en bloc resection were not feasible due to comorbidities and the high-risk clinical status of the patients.

Treatment-related toxicity was assessed retrospectively according to CTCAE version 5.0. Overall, treatment was well tolerated, with only grade 1–2 toxicities reported, including esophagitis, pneumonitis, dyspnea, fatigue, and cough, and no grade ≥ 3 adverse events observed. Treatment and toxicity detailed information are summarized in Table 1.

### Prognosis and outcome

**Fig. 1:**
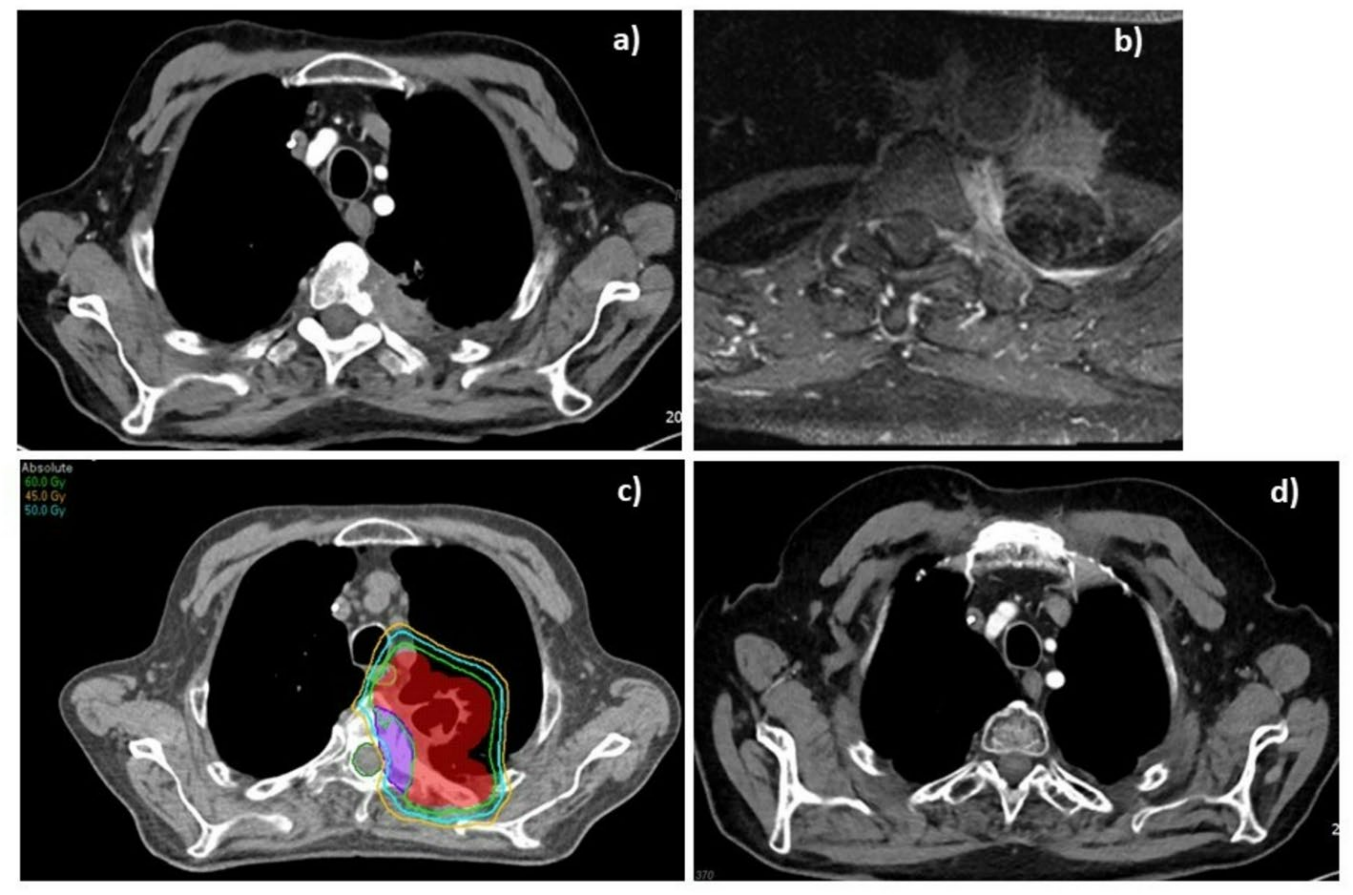
**a** Contrast-enhanced axial computed tomography (CT) and **b** magnetic resonance imaging of a patient with locally advanced non-small lung cancer patients invading the spine before radiotherapy (RT); **c** RT planning with isodose 45 Gy, 50 Gy and 60 Gy, and **d** CT imaging after RT in the follow up obtaining good response

The median overall survival for the entire cohort was 7 months (range 2–26 months). Based on the prognostic scoring system by Lei et al., patients in this series were categorized into two of the three prognostic groups. Four patients (Cases 1, 3, 6, and 7) had low-risk scores (4–5 points) (see Fig. 1). Among the low-risk patients, one achieved prolonged survival of more than two years following radiotherapy, while another showed temporary disease stability. The remaining two low-risk patients died due to local progression, with survival durations of 2 and 7 months.

**Fig. 2:**
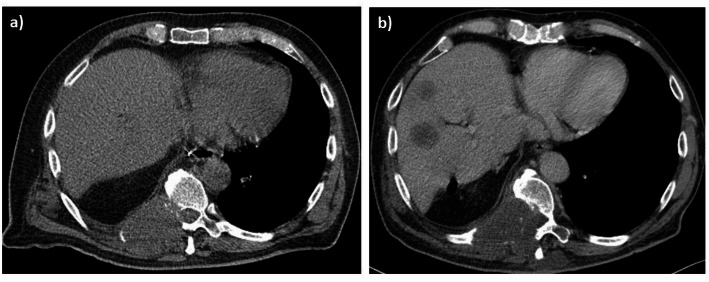
**a** Axial computed tomography (CT) imaging of a patient with locally advanced non-small lung cancer invading the spine before radiotherapy (RT) and **b** contrast-enhanced CT imaging with local and progressive disease after RT in the follow up with poor prognosis

Three patients (Cases 2, 4, and 5) were classified in the intermediate-risk group (6–7 points) (see Fig. 2). All of these patients died during or shortly after treatment due to local and/or distant progression. times in this group ranged from 5 to 13 months. No patients in this cohort fell into the high-risk category (8–10 points) according to the applied scoring criteria. The score points and outcome are summarized in Table 2.

## Discussion

The SWOG 9416 trial (Intergroup 0160) demonstrated that induction chemoradiation followed by surgery resulted in favorable long-term survival outcomes for patients with superior sulcus NSCLC, including those with vertebral and/or spinal invasion (approximately 22% of patients) [14]. Despite the complexity of resection in such cases, selected patients with spinal involvement could still achieve curative outcomes with aggressive multimodal therapy. However, among patients with primary tumor involvement of the spine, 7 patients were deemed inoperable, meaning that approximately half of this patient category could not undergo surgery due to the extent of the disease or other factors. Furthermore, in our multidisciplinary team, radical surgery remains the first choice for operable patients with superior sulcus tumors or tumors invading the spine, even after neoadjuvant therapy, provided that resection is technically feasible and complete resection is achievable.

The wide range of prescribed radiotherapy doses (40–66 Gy) reflected individualized treatment planning based on tumor extent, anatomical constraints, and clinical presentation. Radiotherapy was delivered with a definitive-dose prescription in all patients; however, in several cases it also aimed to provide symptom relief, particularly in the presence of pain or neurological compromise related to spinal involvement. While the primary tumor and involved lymph nodes were treated with definitive doses whenever feasible, lower doses were sometimes prescribed to the involved vertebral regions due to spinal cord dose constraints and the proximity of other critical structures. Consequently, treatment planning required careful balancing of adequate tumor coverage and potential local control with the need to respect spinal cord tolerance and minimize the risk of radiation-induced toxicity [15].

In a recent retrospective study of 16 patients with locally advanced NSCLC invading the spine, all treated with induction CRT followed by en-bloc resection, the authors compared one-step versus two-step surgical approaches [16]. While two-step surgery evolved to reduce perioperative risks, overall morbidity remained high, with 25% experiencing major perioperative and 56.2% major postoperative complications, despite the goal of achieving long-term survival. The 3-year survival data observed in the study by Drevet et al. suggest a potential benefit of two-stage surgery in terms of overall survival (86% versus 40% in the one-stage group).

Our current series reflects the complexity of another scenario regarding spinal invasion in LA-NSCLC. All patients were deemed inoperable due to comorbidities or poor performance status, emphasizing the need for alternative strategies beyond radical surgery. In contrast to most previous reports where radiotherapy was mainly used with palliative intent [8, 17], patients in our series received radiotherapy with curative-dose whenever feasible despite their unfavorable clinical status. While published radiotherapy reports generally describe limited survival outcomes, small surgical series with aggressive multimodal treatment have shown better survival in highly selected patients [10, 16]. Our findings suggest that curative-intent radiotherapy may represent a reasonable treatment option for patients who are not candidates for radical surgery.

A study by Lei et al. developed a prognostic scoring system to guide surgical decision-making in patients with symptomatic metastatic spinal cord compression from NSCLC [13]. Based on retrospective analysis of 64 patients, four preoperative factors—ECOG performance status, number of involved vertebrae, presence of visceral metastases, and time to motor deficit development—were identified as independent predictors of survival. The resulting score stratified patients into three prognostic groups with significantly different 6-month survival rates (95%, 47%, and 11%), offering a practical tool to tailor treatment intensity. Indeed, patients with low scores (4–5) benefited most from radical surgery, while those with high scores (8–10) were more suitable for supportive care or radiotherapy due to poor survival and functional outcomes.

In the original study by Lei et al., the proposed scoring system stratified patients with spinal metastases from NSCLC into prognostic groups with significantly different survival outcomes, underscoring its potential utility in guiding treatment decisions [13]. In our cohort, we retrospectively applied the Lei scoring system to evaluate its prognostic relevance and to explore clinical outcomes across the corresponding risk groups. Our patients were distributed across the low- and intermediate-risk categories, with no individuals classified as high-risk (score 8–10). Among the four patients in the low-risk group (score 4–5), two demonstrated relatively favorable outcomes, with one patient achieving prolonged survival of over two years post-radiotherapy and another exhibiting stable disease at last follow-up.

These findings are consistent with Lei et al.‘s original observations that lower scores are associated with improved prognosis. However, the remaining two patients in this group experienced early mortality due to local progression, suggesting that even within the low-risk category, significant variability in treatment response and disease course exists. This variability points to the need for integrating rapid diagnostic pathways and decision-making frameworks in the management of patients who are non-surgical candidates, to ensure timely submission to appropriate therapies.

Patients in the intermediate-risk group (score 6–7) uniformly had poor outcomes, with all experiencing disease progression and death within a year. However, this group may benefit from more aggressive multimodal approaches or clinical trial enrollment when feasible. While the prognostic score provides a useful framework, our experience suggests that prompt initiation of therapy could be a critical modifier of outcomes, particularly in borderline-risk patients. This distribution may reflect specific selection criteria or referral patterns, as all patients were deemed unsuitable for radical surgical intervention due to comorbidities, or performance status.

Our findings suggest that timely decision-making and early initiation of non-surgical treatments, such as local RT and systemic therapy, may significantly influence outcomes, particularly in patients with lower prognostic scores. On the other hand, our series is limited by the small sample size, the descriptive/retrospective nature of the data and the disease-stage heterogeneity. Due to these important limitations and the rarity of this clinical scenario, our data don not support the absolute treatment effectiveness or survival benefit of radiotherapy. Moreover, the prognostic scoring system proposed by Lei et al. was originally developed for patients with metastatic spinal cord compression rather than direct spinal invasion from primary tumors. Therefore, its applicability in the present cohort should be interpreted with caution.

Although prognosis is typically poor in patients with LA-NSCLC invading the spine, selected patients may benefit from aggressive multimodal therapy and individualized treatment planning and [6–7, 12]. Radiotherapy may contribute to local disease control, symptom relief, and meaningful survival in well selected patients. In the modern treatment era, the integration of radiotherapy with immunotherapy or targeted therapies may further improve outcomes in this challenging clinical setting. Importantly, careful patient selection and treatment in specialized centers with dedicated multidisciplinary teams remain key element in achieving optimal outcomes.

In conclusion, radiotherapy-based multimodal treatment appears feasible in selected patients with advanced NSCLC and spinal infiltration who are not candidates for radical surgery. However, due to the small sample size, the impact on survival remains uncertain and requires validation in larger studies.

## Data Availability

The datasets generated during and/or analysed during the current study are available from the corresponding author on reasonable request.

## Abbreviations

MRI: Brain magnetic resonance imaging
CRT: Chemoradiation
CT: Computed tomography
ECOG: Eastern Cooperative Oncology Group
LA-NSCLC: Locally advanced non-small cell lung cancer
PET-CT: Positron emission tomography – computed tomography
PS: Performance status
RT: Radiotherapy
